# Bromodomain and extra-terminal motif inhibitors: a review of preclinical and clinical advances in cancer therapy

**DOI:** 10.4155/fsoa-2018-0115

**Published:** 2019-01-29

**Authors:** Ali Alqahtani, Khalil Choucair, Mushtaq Ashraf, Danae M Hammouda, Abduraham Alloghbi, Talal Khan, Neil Senzer, John Nemunaitis

**Affiliations:** 1 Department of Internal Medicine, University of Toledo College of Medicine & Life Sciences, Toledo, OH, 43614, USA; 2 Division of Hematology & Medical Oncology, Department of Medicine, University of Toledo College of Medicine & Life Sciences, Toledo, OH, 43614, USA; 3 ProMedica Health System, Toledo, OH, 43606, USA

**Keywords:** BET inhibitors, BET protein, cancer, clinical trials, combination therapy, hematological malignancies, solid tumors, targeted therapy

## Abstract

Histone lysine acetylation is critical in regulating transcription. Dysregulation of this process results in aberrant gene expression in various diseases, including cancer. The bromodomain, present in several proteins, recognizes promotor lysine acetylation and recruits other transcription factors. The bromodomain extra-terminal (BET) family of proteins consists of four conserved mammalian members that regulate transcription of oncogenes such as *MYC* and the NUT fusion oncoprotein. Targeting the acetyl-lysine-binding property of BET proteins is a potential therapeutic approach of cancer. Consequently, following the demonstration that thienotriazolodiazepine small molecules effectively inhibit BET, clinical trials were initiated. We thus discuss the mechanisms of action of various BET inhibitors and the prospects for their clinical use as cancer therapeutics.

## Lay abstract

Addition of acetyl groups to histone proteins that are present in DNA is critical for proper copying of genetic information into producing proteins. This process is dysregulated in various diseases including cancer, resulting in continuous production of oncogenes – genes responsible for promoting cancer – such as *NUT* and *MYC*. Bromodomain extra-terminal (BET) proteins mediate acetyl addition, and as a result, transcription. BET inhibitors are small molecules that block the action of BET proteins and the transcription of oncogenes. In this review, we discuss the different available BET inhibitors, the way they act, and their potential use in clinical settings for cancer treatment.

Acetylation of histone lysine is arguably the most dynamic of the protein post-translational modifications, which participates in structural changes in chromatin and, in addition, regulates various cellular processes, including protein conformations and interactions [1]. Given that histone lysine acetylation has been associated with transcriptional activation [2], one would logically conclude that misregulation of histone acetylation could result in aberrant expression of oncogenes and, consequently, initiation and/or maintenance of cancer [3]. Three types of proteins regulate lysine acetylation and consequent effects, namely, bromodomain (BRD) proteins [4,5]; histone acetyltransferases; and histone deacetylases and sirtuins [6–9]. Acetyltransferases are the ‘writers’ of histone acetylation, and BRDs are the ‘readers’, whereas deacetylases and sirtuins are the ‘erasers’ [9].

The acetyl-lysine-binding BRDs belong to a family of evolutionarily conserved protein modules originally identified in chromatin-associated proteins and are a critical component in the functionality of histone acetyltransferases [10]. BRDs tether those to transcriptional sites and regulate the activities of multi-subunit chromatin remodeling complexes [11], allowing for involvement in diverse mechanisms underlying a range of cellular events [12].

Bromodomain and extra-terminal (BET) proteins belong to the BRD proteins family and share a common domain architecture comprising two N-terminal bromodomains (BD1 and BD2) that interact with acetylated lysine residues on histone tails and an extra-C terminal domain. The BET family consists of four conserved mammalian members, namely, BRD containing 2 (BRD2), BRD3, BRD4 and BRDT [13]. Filippakopoulos *et al*. [14] were the first to characterize a thieno-triazolo-1,4-diazepine histone-binding module inhibitor, JQ1, a cell-permeable small molecule that competitively and specifically binds to the BD1 and BD2 bromodomains with high affinity; this then paved the way for the further development of other BET inhibitors, now being assessed clinically across a range of cancers.

## Biology of BET proteins

**Figure 1.**
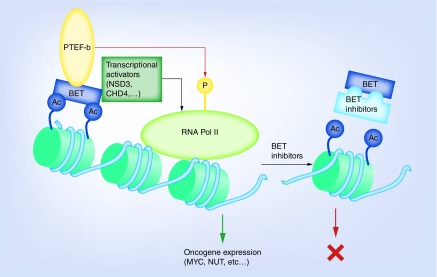
**Biology of bromodomain and extra-terminal inhibitors and their role in cancer therapy.** BET protein binds to acetylated histones and recruits, via its BRD4 domain, PTEF-b to sites of active transcription of growth-promoting genes such as *MYC* and *NUT*. In addition, the ET domain of BRD4 independently recruits transcriptional activators such as NSD3, JMJD6 and CHD4 to further increase rates of transcription. BET inhibitors block the initial binding of BET proteins to acetylated histones, and thus halts the transcriptional cascade of oncogenes. BET: Bromodomain and extra-terminal; PTEF-b: Positive transcriptional elongation factor complex.

Although BRDT is primarily expressed in germ cells, BRD2, 3 and 4 are ubiquitously expressed and interact with acetylated histone tails to regulate transcription. Within the BRD family, sequence variations in the interhelical ZA and BC loops of the distinct structural ‘BRD fold’ result in different protein-binding sites and affinities [15]. BRD4 recruits PTEF-b to sites of active transcription of growth-promoting genes [16]. During elongation, Ser2 of RNA polymerase II is phosphorylated by PTEF-b, which depends on BRD4 for its nuclear localization and activation of its CDK9 kinase subunit [17]. Thus, BRD4 is involved in the conversion of basal transcription to high rates of active elongation by RNA polymerase II (Figure 1). In addition, the ET domain of BRD4 independently recruits transcriptional activators such as NSD3 (a histone methyl transferase), JMJD6 (a histone arginine demethylase) and CHD4 (the catalytic subunit of the NuRD chromatin remodeler) [18]. Unlike BRD4, BRD2 provides a scaffold on chromatin for histone acetyltransferases, histone deacetylases and E2F, thereby coupling histone acetylation to transcription in a PTEF-b-independent manner [16]. BRD3 regulates transcription via the E2F-Rb pathway and also targets GATA1 to chromatin [19].

BET proteins also act as mitotic bookmarks and regulators of the cell cycle. BRD4 remains associated with acetylated H4K5 on chromatin during mitosis, leading to rapid decompaction of adjoining chromatin and transcription [20]. BRD4 marks the transcription start sites of many *M/G1* genes and promotes progression of the cell cycle from G1 to S and G2 to M phases [21,22]. In addition, E2F1 and E2F2, the key transcriptional regulator of S phase, are associated with BRD2 [23].

## BET proteins in cancer

As mentioned previously, altered histone acetylation is associated with aberrant transcription of cancer-related genes. BET proteins promote aberrant expression of the *MYC* oncogene in various hematologic malignancies such as mixed-lineage leukemia, acute myeloid leukemia (AML), Burkitt's lymphoma and Burkitt-like lymphoma; in these cells, disruption of BET binding significantly reduces cellular proliferation and induces apoptosis [24–28]. *MYC* overexpression is also documented in a number of solid tumors including lung, ovary and breast cancer [29]. BRD4 recruits a histone methyltransferase to target genes in ER-positive cells, thus constitutively activating estrogen signaling, a critical pathway in breast cancer tumorigenesis [30]. BRD4 also forms super enhancer complexes with the Mediator complex, a multiprotein transcriptional regulator (containing the CDK8/CDK19/MED12/MED13 kinase module), via which it regulates the expression of oncogenic drivers such as *MYC*, a super-transcription factor deregulated in a majority of cancers [31]. BET proteins are overexpressed in glioblastoma and in primary and metastatic melanoma [32,33]. Consequently, inhibition of BET activity using small-molecule inhibitors might play a significant role in the treatment of these cancers. Furthermore, BET family proteins have been identified in oncogenic rearrangements comprising highly oncogenic fusion proteins that play critical roles in the development of several types of cancer. For example, the BRD-NUT in-frame fusion proteins (primarily t(15;19) involving BRD4) drive carcinogenesis in NUT midline carcinoma (NMC), an aggressive squamous cell carcinoma [34]. Notably, *MYC* is a downstream target of BRD/NUT [35]. Details regarding the involvement of BET proteins in different types of cancer and the efficacy of using BET inhibitors as cancer therapeutics will be discussed below.

## Targeted therapy using BETs

Small-molecule BRD inhibitors were first identified based on structural characterization of the BRD acetyl-binding pocket and nuclear magnetic resonance spectroscopy-based screening of numerous candidate compounds [6,36]. These studies focused on the acetyltransferase CREB-binding protein, which acetylates and modulates p53 tumor-suppressor protein stability and function during DNA damage repair [36,37], and although they identified chemical compounds with low affinity for the BRD pocket and therefore unsuitable for clinical use, they did provide proof-of-principle that BRD inhibition was feasible [37]. Subsequently, multiple small-molecule higher-affinity inhibitors of BETs have been developed. The thienotriazolodiazepines, JQ1 and I-BET, both interact with NF-κB and induce apoptosis in drug-resistant leukemia [38]. I-BET762 mimics acetylated histones to disrupt chromatin complexes. PFI-1 is a highly selective dihydroquinazoline-2-one inhibitor, which blocks the interaction of BET bromodomains with acetylated histone tails. Picaud *et al*. [39] used cocrystal structures to show that the PFI-1 inhibitor acts as an acetyl-lysine mimetic that efficiently occupies the acetyl-lysine binding site in BRD2 and BRD4, which subsequently causes G1 arrest, downregulates MYC and Aurora B kinase, and induces apoptosis. The quinazolone RVX-208 is a BET inhibitor that shows highest selectivity for BD2 of BRD2 and BRD3. We will discuss the mechanism of action and clinical utilities of each of these inhibitors.

## Mechanisms of action of BET inhibitors & preclinical studies

Chemical structures and inhibitory activities in preclinical studies of the various BET inhibitors are summarized in Table 1.

**Table 1.** **Preclinical (*in vitro*) inhibitory activity of bromodomain and extra-terminal inhibitors with their respective clinical structures.**

**BET inhibitor**	**Ki^†^ (nM)**	**IC_50_^¶^ (nM)**	**Chemical structure**
**JQ1**	10.7 ± 1.1	77	

**OTX015**	6.0 ± 0.3	75–650	

**BET-d246**	<1	<10	

**ABBV-075**	1-2.2	<100	

**I-BET 151**	20–100	790	

**I-BET 762**	30.3	35	

**CPI 203**	50–90	37	

**PFI-1**	49 × 10^3^	220	

**RVX-208**^‡^	195	510–600	

**Dinaciclib**^§^	37 × 10^3^	50–90	

^†^to BD2 of BRD4 domain unless otherwise specified.

^‡^to BD2 of BRD3.

^§^to BRDT.

^¶^IC_50_ variation within ranges reflect variability of tested cell lines.

BET: Bromodomain and extra-terminal.

### JQ1: a BRD2/4 inhibitor

#### Hematological malignancies

JQ1, a novel thieno-triazolo-1,4-diazepine, initially identified as a small molecule that strongly inhibits NMC, competitively binds to the acetyl-lysine binding motif effecting significant antiproliferative agent in BRD4-dependent cell lines. Screening of various inhibitors of BRD4-NUT showed higher activity of JQ1 compared with other clinical drugs in NMC, suggesting its translational potential [40].

As mentioned earlier, most common cancers (>50%) exhibit *MYC* deregulation [41]. Several studies have validated c-MYC as a therapeutic target [42–44], including transgenic mouse models where suppression of MYC expression resulted in tumor regression. Thus far, a direct targeting approach has been elusive. However, insofar as *MYC* transcription is associated with local and global changes in histone acetylation [45,46], a feasible, alternative, albeit indirect, way of targeting *MYC* transcription is through alteration of its histone acetylation status using a BET inhibitor (BETi). Significantly, using Raji cells, Mertz *et al*. showed BRD2 and BRD4 release from a site upstream of the P1 *MYC* promoter in conjunction with JQ1-mediated silencing of MYC [27]. In multiple myeloma (MM), characterized by dysregulation of multiple factors due in large part to gene rearrangements and translocations of *MYC* [47], BRD4 was found to be enriched at IgH (immune heavy chain) enhancers rearranged at the *MYC* locus. Furthermore, JQ1 exhibits considerable antiproliferative effect, cell cycle arrest and cellular senescence in three murine models of MM, emphasizing the importance of BET BRD inhibition in MM and in other malignancies with pathological c-MYC activation [48]. As to whether or not genomic alterations are *sine qua non* for BETi effectiveness, there are data demonstrating effective JQ1-mediated MYC silencing in both *MYC* amplified and unamplified cell lines [27]. Indeed, although MYC plays a central oncogenic role in T-ALL, genomic alterations are rarely seen. Rather, MYC overexpression is driven via NOTCH-driven transcriptional or PTEN/AKT/PI3K post-translational changes. Exposure of T-ALL cell lines to JQ1 resulted in downregulation of *MYC* RNA levels and protein expression [49]. The mechanism and effectiveness of BETi on MYC expression is highly cell type specific.

JQ1 can inhibit growth and induce apoptosis of human AML cells, including those expressing *FLT3-ITD* (FMS-like tyrosine kinase 3-internal tandem duplication), a mutant proto-oncogene. Cotreatment of JQ1 and a FLT3 inhibitor, FLT3-TKI, significantly reduces the expression of c-MYC, BCL2 and CDK4/6, while synergistically inducing apoptosis of cultured and primary CD34^+^ human AML blast progenitor cells. Furthermore, cotreatment with JQ1 and the pan-histone deacetylase inhibitor panobinostat synergistically induced apoptosis of FLT3-TKI-resistant cells [50]. Activation of intrinsic pathway caspase 3/7, but not extrinsic pathway caspase 8, after JQ1 treatment indicated the selective functional involvement of the former pathway [24].

The transcription factor STAT5 is constitutively active in most leukemia and drives the expression of genes involved in self-renewal, proliferation and survival. BRD2 is a critical mediator of STAT5 function and JQ1 possibly mediates its effect via STAT-5-dependent pathways. Indeed, JQ1 treatment reduced STAT-5-dependent transcription of heterologous reporters and endogenous genes and showed strong synergy with tyrosine kinase inhibitors (TKI) in inducing apoptosis in leukemia cells [51].

Ott *et al*. showed that JQ1 reduced the viability of B-ALL cell lines with high-risk cytogenetics, such as those with *CRLF2* rearrangements, which are observed in approximately 10% of all B-ALL cases. In these cell lines, the IL7 receptor (IL7R) dimerizes with CRLF2 to drive proliferation and inhibit apoptosis via JAK1, JAK2 and STAT5 signaling. Administration of JQ1 downregulated *MYC* and inhibited BRD4 binding to the *MYC* promoter, while further depleting BRD4 from *IL7R* promoter, thereby downregulating *IL7R* expression and reducing JAK2 and STAT5 phosphorylation. Murine xenografts showed increased survival after JQ1 treatment that was concomitant with MYC suppression and inhibition of STAT5 phosphorylation [28].

As an example of MYC-independent targeting, genome-wide analysis of JQ1-induced transcriptional response in MYC-driven B-cell lymphoma revealed reduced BRD4 occupancy at the IFNγ PD-L1 ligand *Cd274* without affecting MYC occupancy, resulting in a reduction of the *CD274* mRNA level. Combined treatment with JQ1 and PD-L1 antibodies showed synergistic responses in mice with MYC-driven lymphoma [52].

#### Solid tumors

##### Nervous system tumors

Medulloblastoma is the most common malignant brain tumor in children and is characterized by amplification of *MYC*, *MYCN* and *MYCL*. JQ1 treatment significantly reduced cell proliferation and induced apoptosis and senescence in different human medulloblastoma cell lines (HD-BM3, ONS-76 and D-341) and effected inhibition of self-renewal and induction of apoptosis in stem cells [53,54]. JQ1 also reduced the expression of MYC-associated proteins such as Cyclin D1 and E2F1 [55]. Similar results were also observed in human neuroblastoma, where JQ1 targets BRD4 to regulate *MYCN* expression and induce cell death [52].

Glioblastoma multiforme (GBM), both highly aggressive and overwhelmingly lethal, is the most commonly seen brain tumor in adults. Cheng *et al*. reported that JQ1 induced marked G1 cell cycle arrest and apoptosis with significant changes in the expression of important GBM genes; *c-MYC*, *p21CIP1/WAF1*, *hTERT*, *Bcl- 2* and *BclxL*. *In vivo*, orthotopic GBM tumors showed significant growth repression following treatment with JQ1 [56].

Diffuse Intrinsic Pontine Glioma (DIPG) is a fatal common brainstem tumor of children with limited effective treatment options. Owing to high MYCN levels, DIPG was treated sequentially with the bromodomain inhibitor JQ1, which targets MYCN, followed by MRK003, a potent and selective gamma secretase inhibitor that downregulates intracellular NOTCH1. This dual targeting inhibited DIPG growth and induced apoptosis, highlighting the efficacy of this therapeutic regimen [57].

##### Colorectal cancer

Colorectal carcinoma is currently the third leading cause of cancer-related deaths in the USA. The molecular prototype of oncogenic evolution, it arises in a step-wise manner from discrete genetic and epigenetic dysregulations including global hypermethylation (expressed as the CpG island methylator phenotype [CIMP]), which silences suppressor genes in 20% of patients (comprising 7% microsatellite stable tumors and 79% MSI tumors) [58,59]. *CCAT1* is a 2628 nucleotide-IncRNA (long noncoding RNA) that appears to be transcribed off the *MYC* super-enhancer [60]. Although CCAT1 expression does not correlate with CIMP status (i.e., CIMP^+^ vs CIMP^-^), there is a correlation of CCAT1 RNA with BRD4 binding at the CCAT1 super-enhancer site [61] despite the lack of association with MYC expression. Importantly, JQ1 selectively reduces MYC expression in CCAT1 expressing cells. *In vivo*, a recent study by Zhang *et al*. treated the SW480 colon cancer mouse xenografts with JQ-1 [62] and demonstrated a significant reduction in tumor growth, improved mouse survival and induction of apoptosis. Interestingly, the study further showed that inhibition of proliferation was mediated via suppression of Wnt/β-catenin signaling and miR-21.

##### Breast cancer

JQ1 inhibits *BRD3-* and *BRD4-*mediated recruitment of WHSC1 to the ERα gene, thereby suppressing the classic estrogen receptor-α signaling pathway, resulting in the growth suppression of tamoxifen-resistant breast cancer cells in culture [30]. In addition, resistance to second-line everolimus (mTORC1 inhibitor) and exemestane (aromatase inhibitor) in women with ER^+^ breast cancer was shown to occur through upregulation of *MYC* driving mTORC1 resistance [63]. JQ1 restored everolimus sensitivity, and a combination of everolimus and JQ1 led to synergistic growth inhibition in 3D Matrigel cultures and xenograft models [63], suggesting at least partial MYC signaling pathway cooptation of the inhibited mTORC1 pathway.

Similarly, Borbely *et al*. [64] showed that JQ1 inhibits proliferation and induces apoptosis of both triple-negative and ER^+^ breast cancer cells. Treatment with JQ1 or the histone deacetylase inhibitor mocetinostat induced global changes in gene expression, resulting in suppression of genes involved in cell-cycle regulation. Combined treatment of JQ1 and mocetinostat synergistically reduced breast cancer cell viability. Furthermore, the combination treatment increased the expression of ubiquitin-specific protease 17 family of deubiquitinating enzymes, which subsequently attenuated the Ras/MAPK pathway and reduced cell viability [64]. JQ1 also synergistically reduced proliferation of triple-negative breast cancer (TNBC) cells with volasertib, an inhibitor of PLK1 (PLK1 is overexpressed in TNBC) and decreased the expression of stem cell markers [65]. Hypoxia is known to stimulate TNBC by triggering metabolic adaptation, angiogenesis and metastasis. da Motta *et al*. observed that JQ1 modulated the expression of hypoxia-inducible genes, including *CA9* and *VEGF-A*, hindered HIF binding to *CA9* promoter, inhibited TNBC growth and prevented xenograft vascularization [66].

JQ1 exerted antitumor activity in a transgenic mouse model of luminal B human breast cancer. The antitumor phenotype was accompanied by downregulation of oncogenes such as the breast carcinoma-amplified sequence 1 and the PDZ domain-containing 1 [67].

The HER2/ERBB2 oncogene is overexpressed in approximately 25% of breast cancer cases. HER2-positive breast cancer patients are treated with lapatinib, a small-molecule kinase inhibitor that prevents downstream MAPK signaling and AKT activation. However, patients develop resistance to therapy by adapting to lapatinib; this adaptation mechanism has been attributed to phosphorylation of ERBB3, and the subsequent induced expression of IGF1R, DDR1, MET and FGFRs. Stuhlmiller *et al*. [68] showed that growth of HER2-positive breast cancer cells was inhibited by both JQ1 and I-BET762. Treatment of SKBR-3 cells with JQ1 prevented lapatinib-induced phosphorylation and expression of ERBB3. JQ1 also suppressed lapatinib-induced expression of FGFR2, DDR1, IGF1R, pFAK, pSFK and pPKCd across multiple cell lines. Combination treatment with JQ1 and lapatinib enhanced the growth inhibitory effect.

The potent pan-BET bromodomain inhibitor JQ1 was key to delineating mechanism of action and establishing proof of principle of BET inhibition. However, JQ1 has a very short half-life and dose concentrations required to mediate single agent activity are above physiologic safety levels *in vivo* [69]. Furthermore, although its efficacy may be due in part to a considerable lack of discrimination between BD1 and BD2 across the BET family members, this broad target spectrum limits JQ1 specificity, and may narrow its therapeutic window. These considerations, specificity, affinity, potency and pharmacodynamics have led to development of alternative compounds.

### OTX015

OTX015, an oral BET inhibitor targeting BRD2 and 4 [70], is now in Phase Ib clinical trial in patients with selected advanced solid tumors. In preclinical studies, OTX015 was observed to possess antiproliferative effects in lymphoma models and affects MYC, NFKB, JAK/STAT pathways, cell cycle regulation and chromatin structure; it increased HEXIM1 mRNA level in AML and ALL cell lines, which is responsible for sequestering the active form of PTEF-b [70]. Although cytostatic in function, OTX015 induced apoptosis in a genetically distinct subgroup of cells derived from activated B-cell-like diffuse large B-cell lymphoma. OTX015 showed *in vitro* synergism with several anticancer agents, especially with mTOR and BTK inhibitors [66,71] and with azacytidine and panobinostat, to retard the growth of leukemic cell lines [70].

### BET-d246

TNBC remains clinically challenging owing to lack of effective targeted therapy. Recently, BET-d246, using a Proteolysis Targeting Chimera strategy conjugating JQ1 with a ligand for the Cullin-4A ligase, was found to exhibit superior potency and antitumor activity against human TNBC cells. *MCL1* is a critical downstream effector for BET degraders, which synergized with small-molecule inhibitors of BCL-xL in triggering apoptosis. BETd-246, and BETd-260 (an analog), effectively depleted BET proteins in multiple murine xenograft models of human breast cancer tumors and exhibited strong antitumor activities at well-tolerated doses [72].

### ABBV-075

ABBV-075 is a potent and selective BET family bromodomain inhibitor targeting both BDs of BRD2, BRD4 and BRDT that recently entered Phase I clinical trials to evaluate its safety and pharmacokinetics in subjects with advanced solid and hematological tumors. Preclinical evaluations indicated that this compound inhibits proliferation of a range of cancer types, including hematological malignancies and solid tumors. In addition to MYC deregulation, ABBV-075 triggers G1 phase arrest and apoptosis (via the intrinsic pathway) primarily in hematological malignancies, for example, AML, non-Hodgkin lymphoma and MM cells. It exhibits synergy with the BCL-2 inhibitor venetoclax in preclinical models of AML, including xenograft mouse models representing AML, MM and NHL, where ABBV-075 produced efficient antitumor efficacies. Its proapoptotic effectiveness in hematological versus solid tumors may depend on the quantitative levels of BCL2 and Bim as well as the BCL2/Bim ratio [73]. Unlike the lack of *in vitro* synergy between ABBV-075 and azacitidine, cytarabine or bortezomib, xenograft models of AML and MM exhibited enhanced activities of azacitidine and bortezomib with relatively low doses of ABBV-075. In fact, ABBV-075 at 0.25 mg/kg per day, when combined with bortezomib caused a deeper tumor response than bortezomib alone and a longer delay of tumor progression after treatment withdrawal. Similarly, combining azacitidine with ABBV-075 at 0.67 mg/kg per day resulted in a tumor response that was superior to monotherapies of either agent alone [73].

### I-BET151

I-BET151, a pan-BET inhibitor, arrests cell cycle progression and decreases proliferation of GBM cells *in vitro* and *in vivo*. Single molecule sequencing revealed that BET proteins target the GBM-specific IncRNA, *HOTAIR*. I-BET151 decreases *HOTAIR* transcription and increases the expression of other GBM-downregulated noncoding RNAs [32]. I-BET151 also suppressed the Hedgehog activity-dependent growth of medulloblastoma cells, both *in vitro* and *in vivo* [74]. In addition, I-BET151 has been shown to exhibit significant antitumor activity in murine models of NUT midline carcinoma, MM, mixed-lineage leukemia and ALL, lung cancer and malignant brain tumor. In AML with mutations in *NPM1* (encoding nucleophosmin), I-BET151 abrogates the *HOX*-independent transcription program [75], and represses the MYC-dependent program in myeloma, leading to strong antiproliferative effect *in vitro* and *in vivo* [76].

### I-BET762

GSK525762A (I-BET762) was optimized from a chemical hit derived from a phenotypic screen attempting to identify small molecules that enhance *ApoA1* expression. I-BET762 treatment of samples derived from patients with NMC resulted in terminal differentiation and proliferation arrest of malignant cells [77] and significantly reduced *MYC* expression in both prostate cancer cells and a patient-derived tumor model associated with growth retardation and reduction of tumor burden [78]. In neuroblastoma models, I-BET 762 triggered apoptosis via BET inhibition of both MYCN and Bcl-2 [79]. It was also shown to affect myeloma cell proliferation and resulted in a survival advantage in a systemic myeloma xenograft model [75].

### CPI203

CPI203 is an analog of JQ1 with superior oral and intraperitoneal bioavailablity. Mantle cell lymphoma, a type of non-Hodgkin's lymphoma, is characterized by plasmacytic differentiation features such as IRF4 and Blimp-1 upregulation, and is usually treated with bortezomib. The development of bortezomib resistance in mantle cell lymphoma may limit its therapeutic efficacy and result in increased tumorigenicity. Combined treatment of bortezomib-resistant cells with CPI203 and lenalidomide downregulated *MYC*, an IRF4 target, and synergistically induced cell death [80]. Addition of CPI203 to lenalidomide therapy further decreased tumor burden in mice, which was accompanied by simultaneous MYC and IRF4 downregulation and induction of apoptosis [80]. Similar findings were described in *in vitro* and *in vivo* myeloma [81].

### PFI-1

PFI-1, a novel dihydroquinazolinone BET chemical probe, binds to the BET bromodomain in a manner that is chemically distinct from those of previously reported BET inhibitors [39]. Aurora kinases are highly expressed in diverse cancer types and are also frequently upregulated in leukemia [82]. MYC and the critical mitotic entry Aurora A and B isoforms interact in a positive reciprocal feedback loop [39]. PFI-1 downregulates Aurora B kinase in leukemic cells and induces caspase-dependent apoptosis and differentiation. PFI-1 and JQ1 dissociate BRD4 from *HOXA9*, a marker of poor prognosis in patients with AML, and promotes differentiation [83].

### BRD3 inhibitors (RVX-208)

BRD3 is primarily involved in recruiting GATA1 to chromatin in hematopoietic cells and regulating differentiation of erythroid, megakaryocyte and mast cell lineages. BRD3 inhibitors have not been extensively studied. However, pan-BET inhibitors such as JQ1 and I-BET151 have been observed to target BRD3 in NMC and leukemia. RVX-208, a derivative of the plant polyphenol resveratrol, binds to BD2 of BRD3 [84] and upregulates *ApoA1* which plays an important role in hepatocellular carcinoma. Given that JQ1 strongly upregulates *ApoA1* in HepG2 cells, it can be used in combination with RVX-208 as a multitarget inhibitor in hepatocellular carcinoma [85].

### Dinaciclib (BRDT inhibitor)

Dinaciclib, an inhibitor of *CDKs 1, 2, 5* and *9* is in Phase III clinical trial for leukemia [86]. Cocrystallization studies showed interaction with one of the BRDT BDs, providing support for further development of ATP site-directed kinase inhibitors and bromodomain inhibitors [87].

## Clinical trials of BET inhibitors

The safety, tolerability, pharmacokinetics, pharmacodynamics and clinical activity of BET inhibitors are currently being investigated in clinical trials for several cancers, including NMC (OTX015 [NCT02259114, developed by OncoEthix] [71] and GSK525762/I-BET762 [NCT01587703, developed by GlaxoSmithKline]), refractory AML and elastodynamics syndrome ([NCT02308761], lymphoma [NCT01949883 for CPI-0610, developed by Constellation Pharmaceuticals], MM [NCT02157636 for CPI-0610, developed by Constellation Pharmaceuticals] and hematological malignancies [NCT01713582 for OTX015, developed by OncoEthix]). Additional single and combination agent trials in TNBC, ER^+^ breast cancers, small-cell and non-small-cell lung cancers, castration-resistant prostate cancer, pancreatic ductal adenocarcinoma, colorectal cancer, neuroblastoma and MYCN-driven solid tumors are summarized in Table 2.

**Table 2.** **Clinical trials of bromodomain and extra-terminal inhibitors.**

**Target**	**BETi agent**	**Target population**	**Study Phase**	**Sponsor**	**Trial no.**
**BRD2/3/4, BRDT**	ABBV-075	Solid tumors, AML, MM, NSCLC, breast cancer	I	Abbvie	NCT02391480

	FT-1101	AML, MDS	I	Forma Therapeutics/Celgene	NCT02543879

	GSK525762/I-BET762	Hematological malignancies (AML, MM, BCL-2/MYC-driven cohorts NMC, SCLC, NSCLC, CRC, NB, CRPC, TNBC, ER-positive breast cancer	I I/II (open)	GlaxoSmithKline GlaxoSmithKline	NCT01943851 NCT01587703

	INCB057643	Any advanced/recurrent malignancy	I/II	Incyte Corporation	NCT02711137

	ZEN003694	Metastatic CRPC Metastatic CRPC + enzalutamide	I I	Zenith Epigenetics	NCT02705469 NCT02711956

**BRD2/3/4**	OTX015/MK-8628	AML, DLBCL Solid tumors (NMC, TNBC, CRPC, NSCLC) GBM NMC harboring BRD4-NUT, TNBC, NSCLC harboring ALK-KRAS fusion, CRPC, PC AML, DLBCL, ALL, MM	I I Phase II (Withdrawn) I I	Merck/Mitsubishi Tanabe Merck/Mitsubishi Tanabe Merck/Mitsubishi Tanabe OncoEthix GmbH OncoEthix GmbH	NCT02698189 NCT02698176 NCT02296476 NCT02259114 NCT01713582

**BRD2/4**	GSK2820151/I-BET151	Solid tumors	I	GlaxoSmithKline	NCT02630251

**BRD2**	CC-90010	Advanced solid tumors, relapsed/refractory NHL	I	Celgene	NCT03220347

**BRD4**	CPI-0610	Lymphoma MM AML, ALL, CML Peripheral nerve sheath tumors II (not yet open)	I I I I	Constellation Pharmaceuticals/Roche	NCT01949883 NCT02157636 NCT02158858 NCT02986919

	PLX51107	Solid tumors, lymphoma, AML, MDS	I	Plexxicon	NCT02683395

	ABBV-744	AML and prostate cancer	I	Abbvie	NCT02391480

**Undisclosed**	BAY1238097	Solid tumors and lymphoma	I (Terminated)	Bayer	NCT02369029

	BI 894999	Solid tumors and NHL	I	Boehringer Ingelheim	NCT02516553

	BMS-986158	Solid tumors	I/II	Bristol-Myers Squibb	NCT02419417

	GS-5829	DLBCL, peripheral T-cell lymphoma, solid tumors ERþ breast cancer in combination with exemestane or fulvestrant Metastatic CRPC as single agent and in combination with enzalutamide	I I/II I/II	Gilead	NCT02392611 NCT02983604 NCT02607228

	INCB054329	Solid tumors, hematological malignancies	I/II	Incyte Corporation	NCT02431260

	RO6870810/TEN-010	MDS, AML	I	Hoffman-LaRoche	NCT02308761

ALL: Acute lymphoblastic leukemia; AML: Acute myeloid leukemia; BETi: Bromodomain and extra-terminal inhibitor; CML: Chromic myeloid leukemia; CRPC: Castration-resistant prostate cancer; DLBCL: Diffuse large B-cell lymphoma; GBM: Glioblastoma multiforme; MDS: Elastodynamics syndrome; MM: Multiple myeloma; NB: Neuroblastoma; NHL: Non-Hodgkin lymphoma; NSCLC: Non-small-cell lung carcinoma; SCLC: Small-cell lung carcinoma; TNBC: Triple-negative breast cancer.

Trials to date have yielded mixed results. A Phase Ib trial of OTX015 in 47 patients with advanced solid tumors demonstrated four partial responses (PRs) and seven cases of stable disease (SD) for 4–8 months. Toxicities included Grade 3 and 4 thrombocytopenia, Grade 3 anemia and Grade 3 fatigue [88]. In another study, BAY1238097 treatment in eight patients with advanced solid tumors or NHL was stopped because of development of grade 3 headache, vomiting and low-back pain, respectively, at sub-therapeutic doses [89]. Of the four patients enrolled in an OTX015 trial for NMC, two achieved CT confirmed PRs, one of whom showed SUV normalization on PET scan and a third SD all with longer than expected survivals [90].

The seed product JQ1 is not being tested in clinical trials due to its short half-life. Open trials include an Incyte Corporation-sponsored Phase I/II of INCB054329 in subjects with advanced malignancies, and Tensha Therapeutics Phase I, multicenter, open-label study of TEN-010 (structurally similar to JQ1 but with longer half-life) for patients with advanced solid tumors (Table 2). GlaxoSmithKline has a dose escalation study of GSK2820151 in five patients with advanced or recurrent solid tumors, whereas Bayer has a Phase I study of BAY 1238097 (which targets the NFKB/TLR/JAK/STAT signaling pathways, MYC and E2F1-regulated genes, cell cycle regulation and chromatin structure [91] for patients with advanced malignancies). OTX015 has been tested in brain tumors in a nonrandomized, multicenter Phase IIa trial, which tested dose optimization in patients with recurrent GBM following failure of front-line therapy; the trial was terminated after 1 year (Table 2). The results of a first-in-human study of ABBV-075 (Mivebresib) in patients with relapsed/refractory AML have recently been published. Besides showing good tolerability in patients, the preliminary results also show antileukemic effects: bone marrow blast count was 50% of baseline in 4/10 evaluable patients, one patient reached complete remission and the median overall survival for all patients was 3.2 months [92].

## Combination therapy

BET inhibitors have been successfully used in combination with epigenetic inhibitors [49,64] (SAHA, mocetinostat), kinase inhibitors [93], immune checkpoint inhibitors [52] (PD-L1), cell cycle inhibitors [94,95], DNA damage repair agents [96] and chemotherapeutic drugs such as vincristine [49], venetoclax [97], bortezomib [73], azacitidine [73] and lapatinib [68] (Table 3), some of which have been discussed earlier.

**Table 3.** **Combination therapy using bromodomain and extra-terminal inhibitors.**

**Author (year)**	**Combination**	**Model used**	**Outcome**
**BET inhibitor + epigenetic inhibitor** Loosveld *et al*. (2014) [49] Borbely *et al*. (2015) [64] Stratikopoulos *et al*. (2015) [93] Fiskus *et al*. (2014) [50] Bauer *et al*. (2018) [99]	HDAC inhibitor SAHA Mocetinostat Kinase inhibitor PI3K inhibitor + JQ1/MS417 JQ1 + FLT3-TK1 Ponatinib + JQ1/dBET1	*Ex vivo* screening assay Mice xenografted with human primary T-ALLs Breast cancer cells Broad range of cancer cell lines NOD/SCID mice injected with OCIAML3 or MOLM13 cells Colon (HCT116, HT29), breast (MCF-7, SKBR3) and ovarian (A2780, SKOV3) cancer cells	Reduces *MYC* expression Reduced breast cancer cell viability via induction of *ULP17* Combined treatment-induced cell death, tumor regression and clamped PI3K signaling in various cancer cell lines Significantly attenuates the expression of *c-MYC*, *BCL2* and *CDK4/6*, while synergistically inducing apoptosis of cultured and primary CD34^+^ human AML blast progenitor cells Sensitizes colon, breast and ovarian cancer cells to JQ1 and dBET1; combination of these two types of inhibitors increased apoptosis and downregulated *MYC*

**BET inhibitor + cell cycle inhibitor** Tontsch-Grunt *et al*. (2016) [94] Bolin *et al*. (2016) [95]	BI 894999 and CDK9 inhibitors Alvocidib and LDC000067 Unidentified BET and CDK2 inhibitors	Mice injected with MV-4-11, THP-1 and MOLM13 cells Mice transplanted with medulloblastoma	Synergistic inhibition of tumor growth and *MYC* expression Synergistic effect on tumor growth inhibition and improvement of overall survival

**BET inhibitor + immune check point inhibitor** Hogg *et al*. (2017) [52]	JQ1 and PD-L1 antibodies	Mice with Myc-driven lymphoma	Synergistic response in mice with lymphoma

**BET inhibitor + DNA damage repair inhibitors** Muralidharan *et al*. (2016) [96]	RVX2135 and ATR inhibitor AZ20	λ820 and λ2749 murine Myc-induced lymphoma xenografts	Synergistic delay in tumor onset in λ820 xenografts; synergistic WBC reduction and improved survival in λ2749 xenografts

**BET inhibitor + chemotherapeutic drugs** Loosveld *et al*. (2014) [49] Bui *et al*. (2017) [73] Bui *et al*. (2017) [73] Bui *et al*. (2017) [73] Stuhlmiller *et al*. (2015) [68]	JQ1+Vincristine ABBV-075 +Venetoclax ABBV-075 +Bortezomib ABBV-075 + Azacitidine JQ1 + Lapatinib	*Ex vivo* screening assay Mice xenografted with human primary T-ALLs AKM1 AML xenografts AKM1 AML xenografts AKM1 AML xenografts ERRB2-positive breast cancer cell lines resistant to lapatinib	Reduces *MYC* expression Induced apoptosis via modulation of the intrinsic apoptotic pathway, tumor regression Synergistic effect on tumor regression with better tolerance by xenografts than BET inhibitor monotherapy Combination treatment with JQ1 and lapatinib showed stronger growth inhibitory effect of ERRB2-positive breast cancer, reduced levels of ERBB3, IGF1R, DDR1, MET and FGFR

ALL: Acute lymphoblastic leukemia; AML: Acute myeloid leukemia; ATR: Ataxia Telangiectasia Mutated and RAD-3 related ; BET: Bromodomain and extra-terminal; FGFR: FGF receptor; HDAC: Histone deacetylase; MET: Tyrosine-protein kinase; NOD/SCID: Nodulation Factor/Severe Combined Immune Deficiency; SAHA: Suberanilohydroxamic acid; WBC: White Blood Cells.

Resistance to a Class I PI3K inhibitor in a PI3K and MYC-driven model of metastatic breast cancer was associated with feedback activation of tyrosine kinase receptors, AKT, mTOR and MYC. BET inhibitors alone could not control the growth of these tumors. However, combined PI3K and BET inhibition induced cell death, tumor regression and dampened inhibition of PI3K signaling in metastatic breast cancer cell lines as well as in ovarian, colorectal, GBM and prostate cancer cell lines, indicating a strategy for circumventing resistance to kinase inhibitor therapy [93] (Table 3). In this study, the combination of BET inhibition along with PI3K/AKT/PTEN pathway inhibition was shown to maximize the downregulation of *c-myc* expression and to overcome resistance to each of the single agents. In colon cancer cell lines with activating KRAS mutation, inhibition of the p110α subunit of PI3K using a PI3K inhibitor reduced viability and induced apoptosis or cell cycle arrest [98]. These findings altogether suggest that combining BETi and PI3K inhibitor could potentially act synergistically to prevent resistance to kinase inhibition. Furthermore, since the additive effect seems to be mediated via inhibition of signaling across the Ras pathway, the effect could potentially be more amplified by further inhibiting the Ras/MAPK pathway using an MEK inhibitor.

Ponatinib, a multikinase inhibitor that inhibits ABL and FGFR, sensitizes colon, breast and ovarian cancer cells to JQ1 and dBET1. Both single agent ponatinib and BET inhibitors show significant anticancer activity against several hematological malignancies unlike FGFR and/or MYC-driven solid tumors. Interestingly, a combination of the two inhibitors increased apoptosis and downregulated *MYC* [99] (Table 3).

Venetoclax and JQ1 acted synergistically in an *in vitro* drug screening in patient-derived primary leukemia specimens and cell lines (Table 3). This synergism was confirmed *in vivo* using a T-ALL cell line and patient-derived xenograft models. The therapeutic benefit of this drug combination might partially be mediated by acute induction of the proapoptotic factor BCL2L11 and concomitant loss of BCL-2 upon BET bromodomain inhibition (encoded by *BCL2L11*) to BCL-2 [97].

## Resistance to BET inhibitors

Thus far, of the various mechanisms of resistance to BET inhibitors that have been detected, none of them are genetic target aberrations, in other words, *BRD2/3/4* mutations. AML JQ1-resistant cells do not undergo apoptosis but show upregulation of Beclin1, increased LC3 lipidation and formation of autophagosomes. AMPK (p-Thr172)-ULK1 (p-Ser555) pathway activation was associated with JQ1-induced autophagy in the resistant cells independent of mTOR signaling. In support, pharmacological AMPK inhibition or knockdown of PRKAA/AMPKa suppressed autophagy and promoted JQ1-induced apoptosis in AML stem cells [100,101]. Similarly, bortezomib synergistically sensitized BETi-resistant cells to JQ1 treatment, and the JQ1-bortezomib combination induced G2/M arrest in colorectal cancer cells. Although inhibition of NF-κB by bortezomib or NF-κB inhibitor rendered BETi-resistant cells sensitive to JQ1 [102], bortezomib also effects phosphorylation of Bcl-2 and cleavage products that also led to G2/M arrest and apoptosis [103]. Other resistance mechanisms include compensatory upregulation of MYC through the WNT signaling pathway [104,105], hyperphosphorylation of BRD4 due to downregulation of the PP2A phosphatase [106] and increased expression of BCL2L1/BCL-XL [106]. Limits involved in resistance to small-molecule BET inhibitors may also be addressed or compensated via design and mechanism of the actual inhibition (i.e., proteolysis targeting chimera, methyl-acyl-pyrroles) [107]. Further study on a clinical basis, possibly in combination, will be needed to fully determine role of BET inhibitors in oncology.

## Toxicities of clinically used BET inhibitors

A study that directly evaluated the side effects of sustained BET inhibition in the transgenic RNAi mouse model showed that BRD4-depleted mice displayed reversible hyperplasia, alopecia and stem cell depletion in the small intestine. Furthermore, intestines of BRD4-supressed mice showed impaired regeneration following irradiation, suggesting caution regarding the combination of BETi with radiation therapy [108]. In current clinical trials, more commonly seen toxicities are gastrointestinal (diarrhea, nausea, vomiting), fatigue, hyperbilirubinemia, thrombocytopenia and anemia.

In the first-in-human clinical trial (NCT02391480) for assessing the safety and pharmacokinetics of ABBV-075 in patients with advanced tumors, 71 patients (98.6%) reported ≥1 treatment-emergent adverse events (TEAEs); thrombocytopenia (56.9%), dysgeusia (48.6%), fatigue (43.1%) and nausea (34.7%) were the common side effects. Grade 3/4 TEAEs were reported in 52 patients (72.2%), among which thrombocytopenia and anemia were most common. Dose-limiting toxicities (DLTs) included thrombocytopenia, fatigue and elevation in aspartate aminotransferase levels, gastrointestinal bleeding and hypertension [92].

In the clinical trial NCT02516553 for BI894999, 21 patients with advanced solid tumors were treated with six dose levels of the drug daily (Arm A), whereas seven patients were administered intermittently with two dose levels of the drug (Arm B) for 3 weeks. The maximum tolerated dose was 1.5 mg in Arm A. Dose escalation was discontinued in Arm B because of cardiac changes in three, and elevated serum troponin level of eight patients. The most frequent (≥10%) TEAEs were fatigue, thrombocytopenia, decreased appetite, diarrhea, increased troponin T, dysgeusia, nausea, stomatitis, neutropenia and vomiting. DLTs included thrombocytopenia grade (G) 4, increased troponin (G3), hypophosphatemia G3 and multiple G2 events in one patient preventing adequate dose intensity in cycle 1. Thrombocytopenia prevented continuous dosing [109].

In the first-in-human Phase I study of CPI-0610 in patients with relapsed or refractory lymphomas (NCT01949883; n = 64), the most common TEAEs were thrombocytopenia, fatigue, nausea, decreased appetite and anemia. However, the thrombocytopenia was reversible and not cumulative. Nonetheless, five patients showed objective response, which included two complete responses (CRs) and three PRs; five patients had prolonged (>6 months) SD, indicating that CPI-0610 was a well-tolerated drug with clinical activity in patients with advanced lymphoma [110].

OTX015 (MK-8628) was clinically tested in a Phase IIa trial (NCT02296476) in patients with recurrent GBM. A traditional 3+3 dose escalation scheme was followed. Patients with first recurrence of GBM were orally administered MK-8628 at three dose levels (DL1: 80 mg QD, DL2: 120 mg QD, DL3: 160 mg QD) with 4-week cycles; the effects of the drug on the tumor was assessed using MRI after every two cycles. Three patients showed DLTs – one at DL1 (G3 thrombocytopenia >7 days) and two at DL3 (G3 thrombocytopenia >7 days without bleeding; G3 hyperbilirubinemia for 2 days). Other DLTs included G3 thrombocytopenia, G1-2 diarrhea and G2 myalgia. Overall, the drug was well-tolerated. However, owing to lack of detectable clinical activity the trial was close [111].

OTX015 was also tested clinically in a Phase I trial (NCT01713582) in patients with AML who had previously received two other lines of therapy. Six dose levels were tested and patients were administered one dose daily. No DLT was recorded until 160 mg/day, when one patient had G3 diarrhea and another had G3 fatigue. Common toxic effects for all OTX01 doses were fatigue (including G3 in three patients) and elevation in bilirubin concentration (including G3-4 in two patients). Nonetheless, three patients (receiving 40, 80 and 160 mg/day) achieved CR or CR with incomplete recovery of platelets lasting 2–5 months, and two additional patients had partial blast clearance [112]. Similarly, OTX015 was tested in another Phase I clinical trial involving patients with lymphoma and myeloma. In this trial, 80 mg once daily was selected as the tolerable dose as higher doses or multiple lower doses resulted in DLTs such as thrombocytopenia, anemia, neutropenia, diarrhea, fatigue and nausea [113].

A first-in-man 3+3 dose escalation study of PLX51107 (20–160 mg QD) in adult patients with relapsed or refractory solid tumors and AML was conducted to determine the recommended Phase II (NCT02683395). The most common toxicities (mainly G1-2) in ≥15% of patients included fatigue, vomiting, diarrhea, nausea, hyperbilirubinemia and increase in internal normalized ratio. Three TEAEs (one each of G3 nausea, G2 vomiting and G2 kidney injury) were observed, and patients with extensive hepatic metastases (>50% of the liver) demonstrated more TEAEs. DLT was observed at 20 mg (one G3 thrombocytopenia), 120 mg QD (one G3 nausea) and 160 mg QD (one G2 kidney injury) [114].

The Phase I trial of BAY 1238097 was characterized by the occurrence of two DLTs at the 80 mg/week dose level and all eight subjects included in the safety set experienced at least one TEAE. Six out of eight (75%) subjects had at least one serious adverse event, including at least one treatment-emergent event. Hence, this trial was discontinued (Bayer Clinical Study Synopsis, 2016).

## Conclusion & future perspective

The increased understanding of the effect of genomic and epigenomic aberrations on the transcriptional process has led to a greater appreciation of the role of transcriptional deregulation in the initiation and maintenance of cancer [115]. Promoters regulate transcriptional gene activity. Sequences of lineage-associated transcription factors assemble as gene enhancers and function as an autoregulatory loop. Clusters of these enhancers processing cell identity and/or subserving cellular function are characterized as super-enhancers, which appear to have higher densities of Mediator (MED1) and BRD4 binding [13], particularly those that are oncogenic. Of further importance, although an oncogene may be common to multiple tumor types, the associated super-enhancers can be tumor-type specific. Thus, the super-enhancers play a major role in determining the cell-type-specific response to BETi.

Inhibitors of BET proteins are being extensively studied and many are currently in clinical trial such as OTX015 for hematological cancers and I-BET762 for NMC. With exception, single agent BETis have shown limited long-term effectiveness and multiple mechanisms of resistance have already emerged. Further investigations are necessary to expand our understanding of the function of the various isoforms of each BET protein (produced via alternative splicing), the differences in the expression levels of the BET proteins in different cancer types, and the lineage-related and contextual functionality of the BET-containing tumor molecular elements. The outcome of these investigations will contribute to individualization of therapy to maximize therapeutic effectiveness, minimize inefficient and costly (in terms of patient time and financial investment) application (i.e., by maximizing targeted population/candidate population) and contribute to a logical, fact-based and personalized approach to combinatory and combined modality therapy.

**Executive summary**

- The interaction between genomic and epigenetic aberration in cancer has led to investigation of transcriptional regulation as a potential therapeutic approach in treating malignancies.
- Bromodomain and extra-terminal (BET) proteins are enriched at promoter sites, particularly upstream of oncogenes, where they recruit other transcriptional factors.
- BET inhibitors disrupt this interaction and, through several preclinical studies, were shown to halt transcription of oncogenes, decrease cancer cell survival and induce apoptosis. Many trials are ongoing to further characterize the relevance of BET inhibitor use in a clinical setting.
- Currently, and despite a relatively tolerable safety profile, clinical use of BET inhibitors monotherapy seems to be hindered by apparent resistance, mostly mediated by the emergence of alternative molecular pathways and pathway cross-talk.
- Several studies have successfully established proof of concept for the potential use of BET inhibitors in combination therapy. Namely, combining epigenetic inhibition of BET with inhibition of relevant molecular pathways may not only contribute to clinical efficacy but also pave the way to a personalized approach to combined therapy involving BET inhibitors.
